# Selection of candidates for surgery as local therapy among early-stage small cell lung cancer patients: a population-based analysis

**DOI:** 10.1186/s40880-018-0272-5

**Published:** 2018-03-31

**Authors:** Kaiqi Jin, Kaixuan Zhang, Feng Zhou, Jie Dai, Peng Zhang, Gening Jiang

**Affiliations:** Department of Thoracic Surgery, Shanghai Pulmonary Hospital, School of Medicine, Tongji University, No. 507 Zhengmin Road, Shanghai, 200433 P. R. China

**Keywords:** Small cell lung cancer, Surgery, Local therapy, Radiotherapy

## Abstract

**Background:**

Surgery and radiotherapy are considered local therapies for small cell lung cancer (SCLC). The present study aimed to select candidates for surgery as local therapy among patients with stage I or II SCLC, based on the eighth edition of the TNM classification for lung cancer.

**Methods:**

Patients diagnosed with SCLC between 2004 and 2013 were selected from the Surveillance, Epidemiology, And End Results database. The TNM stage of SCLC in these patients was re-classified according to the eighth edition of the TNM classification for lung cancer. Patients with stage I or II SCLC were included in the present study. Overall survival (OS) and lung cancer-specific survival (LCSS) were separately compared in the different TNM stages between patients who received surgery and radiotherapy as local therapy. Multivariate analysis was applied to evaluate multiple factors associated with survival.

**Results:**

Among the 2129 patients included in the present study, 387 (18.2%) received surgery, 1032 (48.5%) underwent radiotherapy as local therapy, 154 (7.2%) underwent surgery and radiotherapy, and 556 (26.1%) did not undergo either surgery or radiotherapy. Among patients with T1-2N0 (tumor size ≤ 50 mm without positive lymph nodes) disease, patients who underwent surgery had higher 5-year OS and LCSS rates than patients who received radiotherapy (T1N0: 46.0% vs. 23.8%, *P* < 0.001, and 58.4% vs. 36.4%, *P* < 0.001, respectively; T2N0: 42.6% vs. 24.7%, *P* = 0.004, and 48.8% vs. 31.3%, *P* = 0.011, respectively). Multivariate analysis results revealed that surgery was associated with low risk of death. However, among T3N0 or T1-2N1 (stage IIB) SCLC patients, patients who underwent surgery did not have higher 5-year OS and LCSS rates than patients who received radiotherapy (T3N0: 16.2% vs. 26.5%, *P* = 0.085, and 28.7% vs. 30.9%, *P* = 0.372, respectively; T1-2N1: 20.3% vs. 29.0%, *P* = 0.146, and 25.6% vs. 35.5%, *P* = 0.064, respectively).

**Conclusions:**

Based on the assumption that the overwhelming majority of stage I or II SCLC patients who underwent surgery or radiotherapy also received certain types of systemic therapy, only patients with T1-2N0 SCLC may benefit from surgery as local therapy. Patients with T3N0 or T1-2N1 SCLC may consider radiotherapy as local therapy.

## Background

Lung cancer has become the most frequently diagnosed cancer in China [1], and the first leading cause of cancer-related death worldwide [2]. Small cell lung cancer (SCLC) accounts for 10%–20% of lung cancers, in general [3], and it has been estimated that 31,000 new SCLC cases will occur in the United States in 2017 [4]. Due to the nature of rapid growth and early metastasis, SCLC is usually associated with a poor overall prognosis, with the median survival ranging from 2 to 4 months when left untreated [5]. SCLC is highly sensitive to initial chemotherapy and radiotherapy. However, most patients relapse and become resistant to subsequent therapies, and eventually die [6].

Treatment strategies for SCLC have changed a lot in history. Before the 1970s, surgery was performed to treat SCLC. In 1973, the first and only prospective, randomized trial that compared surgery with radiotherapy was conducted by the Medical Research Council (MRC) [7]. The trial revealed that patients treated with surgery had shorter survival than those treated with radiotherapy, and SCLC was considered not suitable for surgery in the latter two decades. A meta-analysis has proved that thoracic radiotherapy combined with chemotherapy moderately prolonged survival in patients with limited SCLC in 1992 [8]. After that, the combination of chemotherapy and radiotherapy became the standard treatment of limited SCLC.

In contrast with the conclusion of the MRC [7], it was also reported that patients with early-stage disease might benefit from surgery [9–18]. The present National Comprehensive Cancer Network (NCCN) guidelines [19] recommends pulmonary resection (lobectomy preferred) and mediastinal lymph node dissection or sampling as the initial treatment and systemic chemotherapy as the adjuvant treatment for patients who have clinical T1-2N0 (seventh edition TNM classification, tumor size ≤ 70 mm without positive lymph nodes) SCLC with negative pathologic mediastinal staging. Patients with limited stage SCLC in excess of T1-2N0 should undergo systemic therapy with or without radiotherapy [19]. It could be concluded that NCCN recommends systemic therapy with or without local therapy (surgery or radiotherapy) for limited stage SCLC and surgery as local therapy due to its suitability for patients with T1-2N0 disease. However, this guideline was based on lower-level evidence, which was considered in category 2A. In fact, the criteria of patients who receive surgery as part of multimodality treatment have varied widely in literature [9–18]. Appropriate candidates for surgery as local therapy remain debatable.

The Surveillance, Epidemiology, and End Results (SEER) database comprises of a set of geographically defined, population-based central cancer registries in the United States that collects data concerning the demographics, diagnosis, treatment and survival outcomes of individual patients. The eighth edition of the TNM classification has been published in late 2016, and the continued usage of this system for SCLC was recommended [20, 21]. The present study aimed to select candidates for surgery as local therapy among early-stage (stage I or II) SCLC patients by analyzing data obtained from the SEER database based on the eighth edition of the TNM classification.

## Methods

### Study cohort

The Incidence-SEER 18 Registries Research Data and Hurricane Katrina Impacted Louisiana Cases were used for the present analysis. Patients who were older than 18 years and diagnosed with primary SCLC between 2004 and 2013 were selected from the SEER database. SCLC was defined by morphology codes 8002 and 8041–8045 and morphology site “lung and bronchus” when using SEER*Stat version 8.3.2. The TNM stage was re-classified according to the eighth edition of the TNM classification for lung cancer [21]. Exclusion criteria included (a) status of surgery or status of radiotherapy could not be identified, (b) cases with an autopsy or death certificate, and (c) the TNM stage could not be re-classified according to the eighth edition of the TNM classification [21].

### Staging

Pathological staging was used for patients who received surgical staging of the mediastinum, whereas the other patients without surgical staging of the mediastinum were clinically staged. The T category for patients was re-classified using the SEER code “CS tumor size,” “lung pleural elastic layer invasion PL by hand or elastic,” “lung separate tumor nodules ipsilateral lung,” and “CS extension,” according to the eighth edition of the TNM classification for lung cancer [21]. The original N and M categories of patients used in the SEER database were reserved and directly transferred into the eighth edition of the TNM classification (except that some specific M1 categories of some patients in the sixth edition were transferred to T4M0 category in the eighth edition) due to the slight difference in N and M categories through the sixth to eighth edition of the TNM classification.

### Groups and stratums

Patients were divided into four groups according to the type of local therapy they received: surgery, radiotherapy, surgery + radiotherapy, and no surgery or radiotherapy. Patients who underwent beam radiotherapy were identified as patients who underwent radiotherapy.

Stage I or II SCLC patients were included in the present study. These patients were divided into four stratums: T1N0, T2N0, T3N0, and T1-2N1. Analysis was separately performed for these four different stratums.

### Outcomes

The outcomes in the present study included overall survival (OS) and lung cancer-specific survival (LCSS), based on SEER codes “Vital status recode study cutoff used” and “SEER cause-specific death classification”, respectively. OS was defined as the time from diagnosis until death or the last follow-up. Patients who were not deceased were censored at the date they were last known to be alive. LCSS was defined as the time from diagnosis until death attributed to lung cancer or the last follow-up, and patients who were not deceased or died due to other causes (not lung cancer) was censored at the date they were last known to be alive or the date they died due to other causes. Patient outcome was achieved up to December 31, 2013.

### Statistical analyses

Continuous variables were compared using the Student’s *t* test. Unordered categorical variables were analyzed using Person’s χ^2^ test, and ordered categorical variables by Mann–Whitney test. Survival curves were constructed using the Kaplan–Meier method and compared using the log-rank test. Multivariable Cox regression models were used to identify relevant variables that affect survival. A two-sided *P* value < 0.05 was considered statistically significant. Statistical analysis was performed using SPSS 23.0 (SPSS Inc. Chicago, IL, USA), and the survival curves were drawn using GraphPad Prism 6.0 (GraphPad Software, San Diego, CA, USA).

## Results

### Baseline characteristics

A total of 2129 patients with stage I or stage II SCLC were included in the present cohort. The numbers of patients with stage IA, IB, IIA, and IIB SCLC were 723, 397, 201, and 808, respectively. The most common local therapy was radiotherapy (*n* = 1032, 48.5%), followed by no surgery or radiotherapy (*n* = 556, 26.1%). Patients who received surgery had a lower TNM stage (*P* < 0.001) and had a higher likelihood of being white race (*P* = 0.011) than patients who underwent radiotherapy. Details of the baseline characteristics of patients are listed in Table 1.

**Table 1:** Baseline characteristics of 2129 patients with SCLC

Characteristic	All patients (*n* = 2129)	Surgery (*n* = 387)	RT (*n* = 1032)	*P* ^b^	Surgery + RT (*n* = 154)	No surgery or RT (*n* = 556)
Age (years)^a^	68.3 ± 9.9	67.8 ± 9.4	67.6 ± 9.7	0.837	63.8 ± 8.5	71.3 ± 10.2
Race				0.011		
White	1875 (88.1)	355 (91.7)	886 (85.9)		146 (94.8)	488 (87.8)
Black	173 (8.1)	22 (5.7)	94 (9.1)		6 (3.9)	51 (9.2)
Others	81 (3.8)	10 (2.6)	52 (5.0)		2 (1.3)	17 (3.1)
Sex				0.965		
Male	988 (46.4)	179 (46.3)	476 (46.1)		66 (42.9)	267 (48.0)
Female	1141 (53.6)	208 (53.7)	556 (53.9)		88 (57.1)	289 (52.0)
Laterality of tumor location				0.740		
Left lung	927 (43.5)	169 (43.7)	451 (43.7)		68 (44.2)	239 (43.0)
Right lung	1191 (55.9)	217 (56.1)	575 (55.7)		85 (55.2)	314 (56.5)
Others	11 (0.5)	1 (0.3)	6 (0.6)		1 (0.6)	3 (0.5)
Surgery method^c^				0.327^d^		
Pneumonectomy	–	11 (2.8)	–		2 (1.3)	–
Lobectomy	–	271 (70.0)	–		102 (66.2)	–
Sub-lobectomy	–	103 (26.6)	–		50 (32.5)	–
T category				< 0.001		
T1	1001 (47.0)	233 (60.2)	441 (42.7)		89 (57.8)	238 (42.8)
T2	839 (39.4)	133 (34.4)	421 (40.8)		52 (33.8)	233 (41.9)
T3	289 (13.6)	21 (5.4)	170 (16.5)		13 (8.4)	85 (15.3)
N category				< 0.001		
N0	1610 (75.6)	324 (83.7)	736 (71.3)		96 (62.3)	454 (81.7)
N1	519 (24.4)	63 (16.3)	296 (28.7)		58 (37.7)	102 (18.3)
TNM stage				< 0.001		
T1N0	723 (34.0)	198 (51.2)	283 (27.4)		56 (36.4)	186 (33.5)
T2N0	598 (28.1)	105 (27.1)	283 (27.4)		27 (17.5)	183 (32.9)
T3N0	289 (13.6)	21 (5.4)	170 (16.5)		13 (8.4)	85 (15.3)
T1-2N1	519 (24.4)	63 (16.3)	296 (28.7)		58 (37.7)	102 (18.3)

*SCLC* small cell lung cancer, *RT* radiotherapy

^a^These data are presented as mean ± standard deviation (SD); other data are all presented as the number of patients, followed by the percentage in parentheses

^b^Comparisons between surgery and radiotherapy groups, except for the comparison of the surgery method

^c^For patients who underwent surgery, surgery information was unavailable for two patients

^d^Comparisons between surgery and surgery + RT groups

### Survival analysis and multivariable Cox regression analysis for the entire cohort

**Fig. 1:**
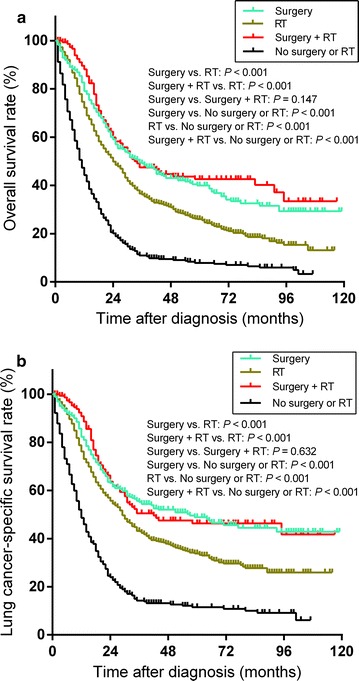
Overall survival (**a**) and lung cancer-specific survival (**b**) for patients with stage I or II small cell lung cancer (SCLC) who underwent surgery, radiotherapy (RT), surgery + RT, or no surgery or RT

The median OS and 5-year OS rate for the entire cohort were 20.0 months and 24.6%, respectively, and the median LCSS and 5-year LCSS rate were 23.0 months and 31.9%, respectively. The median OS and 5-year OS rates for patients who received surgery, radiotherapy, surgery + radiotherapy, and no surgery or radiotherapy were 32.0 months and 38.9%, 24.0 months and 25.9%, 34.0 months and 42.7%, and 9.0 months and 7.2%, respectively. The median LCSS and 5-year LCSS rates for patients who received surgery, radiotherapy, surgery + radiotherapy, and no surgery or radiotherapy were 56.0 months and 48.3%, 29.0 months and 33.8%, 42.0 months and 46.5%, and 11.0 months and 11.0%, respectively. Patients who received surgery with or without radiotherapy had longer OS and LCSS than patients who underwent radiotherapy alone (all *P* < 0.001). When comparing OS (*P* = 0.147) and LCSS (*P* = 0.632) between patients who received surgery alone and surgery + radiotherapy, no significant differences were observed. Patients who received radiotherapy had longer OS (*P* < 0.001) and LCSS (*P* < 0.001) than patients who did not undergo surgery or radiotherapy (Fig. 1). The multivariable Cox regression analysis supported the outcomes of the survival analysis (Table 2).

**Table 2:** Multivariable analysis for overall survival and lung cancer-specific survival of patients with stage I or II SCLC

Variable	Overall survival		Lung cancer-specific survival	
	Hazard ratio (95% CI)	*P*	Hazard ratio (95% CI)	*P*
Age, per year increase	1.031 (1.025, 1.036)	< 0.001	1.029 (1.023, 1.036)	< 0.001
Year of diagnosis, per year later	0.963 (0.943, 0.983)	< 0.001	0.958 (0.936, 0.980)	< 0.001
Race				
White	Reference		Reference	
Black	0.840 (0.687, 1.026)	0.087	0.855 (0.688, 1.063)	0.158
Others	0.951 (0.715, 1.265)	0.730	0.967 (0.708, 1.320)	0.832
Sex				
Male	Reference		Reference	
Female	0.941 (0.847, 1.046)	0.262	0.965 (0.859, 1.084)	0.544
Laterality of tumor location				
Left lung	Reference		Reference	
Right lung	1.010 (0.908, 1.123)	0.860	1.039 (0.924, 1.168)	0.524
Others	1.044 (0.518, 2.102)	0.905	0.793 (0.328, 1.918)	0.607
T category				
T1	Reference		Reference	
T2	1.237 (1.104, 1.387)	< 0.001	1.311 (1.155, 1.488)	< 0.001
T3	1.418 (1.197, 1.680)	< 0.001	1.644 (1.371, 1.972)	< 0.001
N category				
N0	Reference		Reference	
N1	1.330 (1.171, 1.511)	< 0.001	1.410 (1.227, 1.621)	< 0.001
Treatment				
Surgery	Reference		Reference	
RT	1.256 (1.071, 1.475)	0.005	1.265 (1.055, 1.515)	0.011
Surgery + RT	0.860 (0.660, 1.120)	0.263	0.947 (0.710, 1.264)	0.713
No surgery or RT	2.964 (2.509, 3.502)	< 0.001	3.171 (2.631, 3.822)	< 0.001

*SCLC* small cell lung cancer, *CI* confidence interval, *RT* radiotherapy

### Survival analysis between patients who underwent surgery and radiotherapy for each stratum

**Fig. 2:**
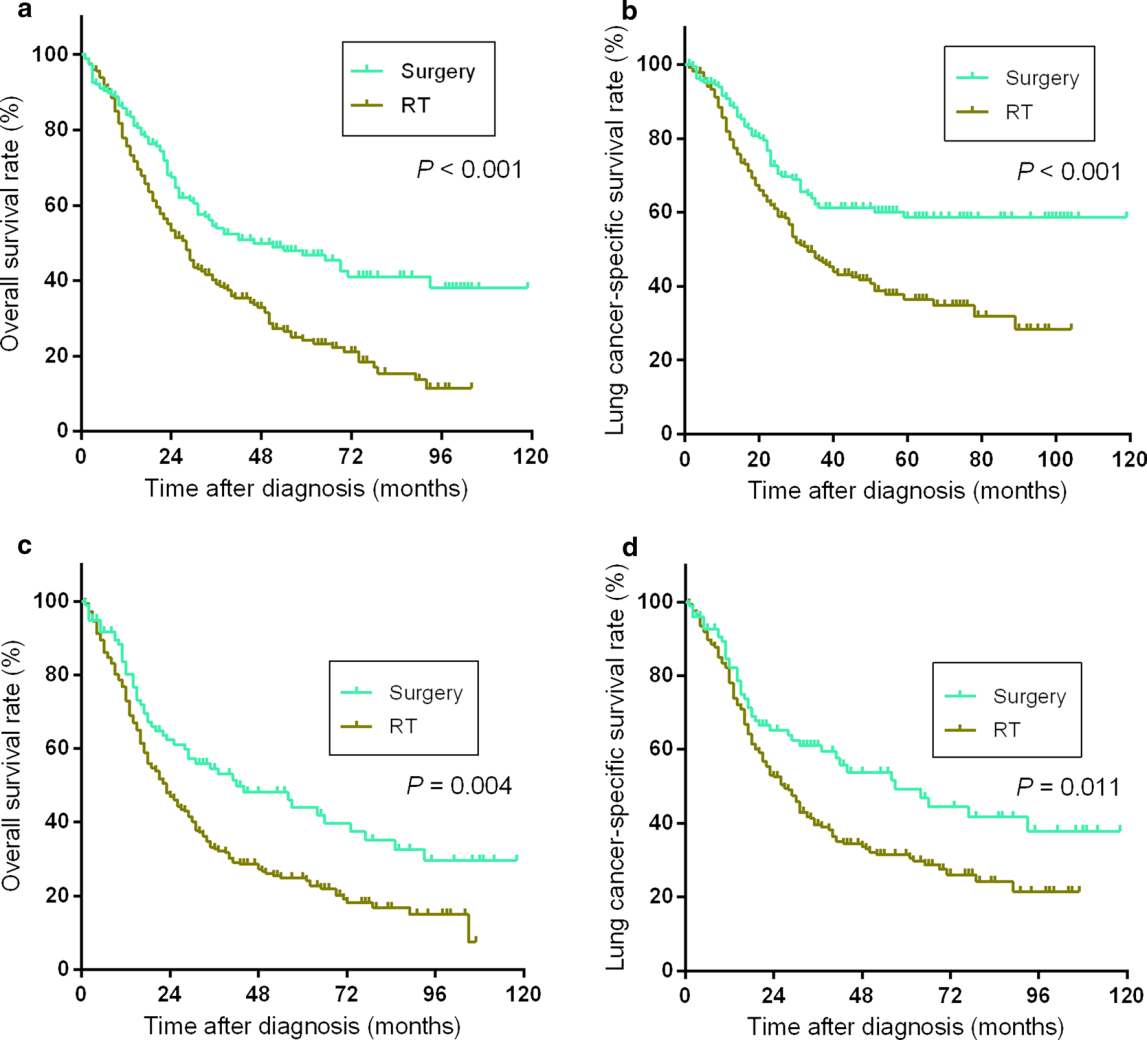
Overall survival (**a**) and lung cancer-specific survival (**b**) for patients with T1N0 SCLC who underwent surgery or radiotherapy (RT) and overall survival (**c**) and lung cancer-specific survival (**d**) for patients with T2N0 SCLC who underwent surgery or RT

**Fig. 3:**
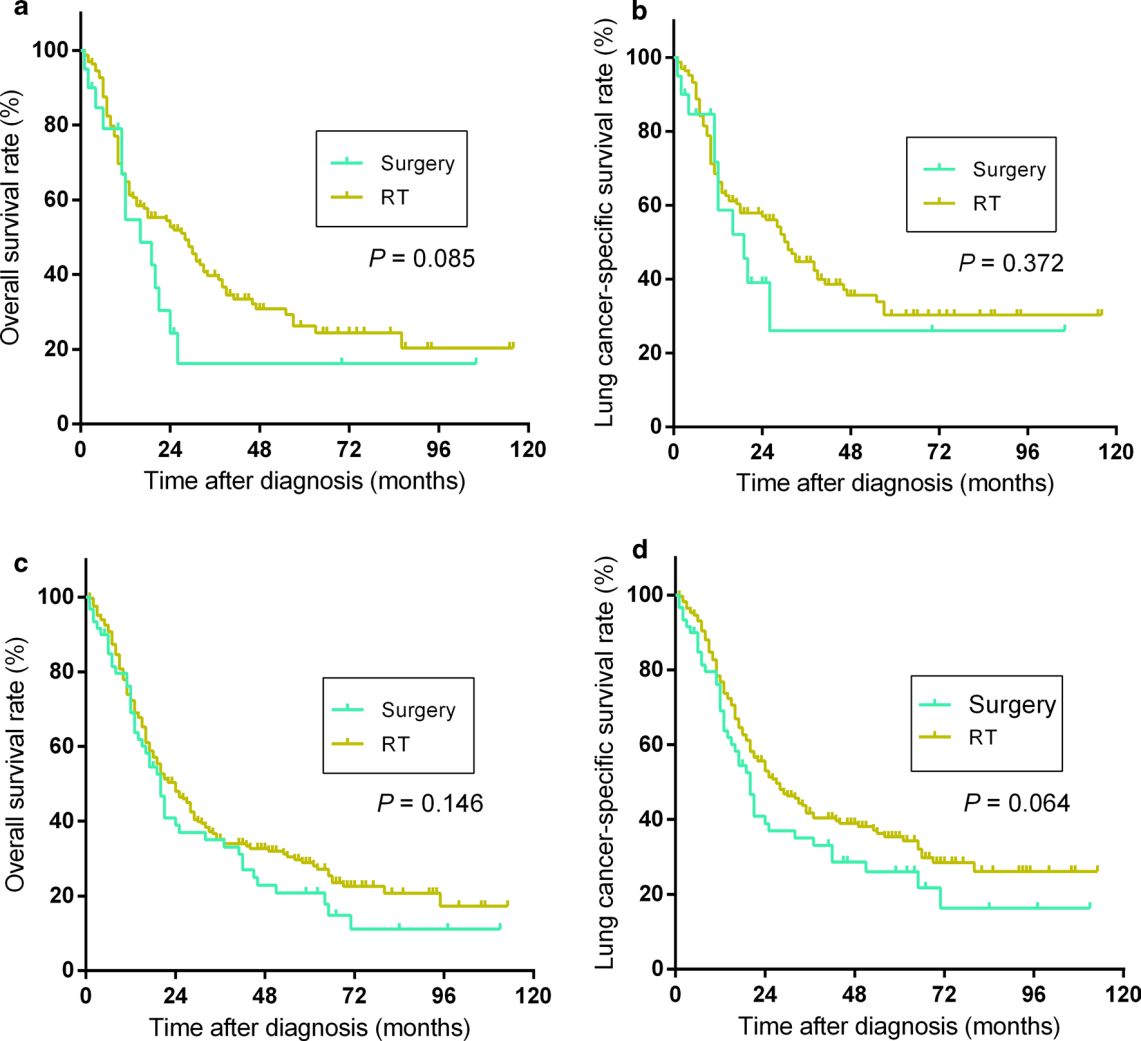
Overall survival (**a**) and lung cancer-specific survival (**b**) for patients with T3N0 SCLC who underwent surgery or radiotherapy (RT) and overall survival (**c**) and lung cancer-specific survival (**d**) for patients with T1-2N1 SCLC who underwent surgery or RT

For T1N0 cases, patients who underwent surgery had longer OS and LCSS than did those who underwent radiotherapy (both *P* < 0.001); 5-year OS rate for surgery or radiotherapy were 46.0% vs. 23.8%, and 5-year LCSS rate for surgery or radiotherapy were 58.4% vs. 36.4%, respectively (Fig. 2a, b). For patients with T2N0 SCLC, similar outcomes of survival analysis were found (Fig. 2c, d); median survival time and 5-year survival rate for patients who underwent surgery or radiotherapy were 41.0 months and 42.6% vs. 23.0 months and 24.7% (*P* = 0.004, OS) and 57.0 months and 48.8% vs. 27.0 months and 31.3% (*P* = 0.011, LCSS). For T3N0 or T1-2N1 cases, patients did not benefit from surgery, compared with radiotherapy. For patients with T3N0 SCLC (Fig. 3a, b), median OS time and 5-year OS rate were 16.0 months and 16.2% in surgery group vs. 28.0 months and 26.5% in radiotherapy group (*P* = 0.085). Median LCSS time and 5-year LCSS rate were 19 months and 29% in surgery group vs. 31 months and 31% in radiotherapy group (*P* = 0.372). For patients with T1-2N1 SCLC (Fig. 3c, d), median survival time and 5-year survival rate of surgery group and radiotherapy group were 20.0 months and 20.3% vs. 24 months and 29.0% (*P* = 0.146, OS) and 20.0 months and 25.6% vs. 27.0 months and 35.5% (*P* = 0.064, LCSS).

### Multivariable Cox regression analysis for each stratum

After adjusting for age, race, sex, laterality of tumor location, and year of diagnosis, the multivariate regression analysis results for each stratum revealed that the patients who underwent surgery had a lower risk of death than patients who underwent radiotherapy in terms of OS (HR: 0.622, 95% confidence interval [CI] 0.481–0.804 [T1N0]; HR: 0.625, 95% CI 0.460–0.849 [T2N0]) and LCSS (HR: 0.600, 95% CI 0.442–0.814 [T1N0]; HR: 0.623, 95% CI 0.445–0.873 [T2N0]) for patients with T1N0 or T2N0 SCLC; for T3N0 cases, surgery was not associated with a low risk of death in terms of OS and LCSS; for patients with T1-2N1 SCLC, surgery was associated with a high risk of LCSS (HR: 1.419; 95% CI 1.003–2.006) (Table 3).

**Table 3:** Multivariable analysis for surgery versus radiotherapy in patients with each stage of SCLC

TNM stage	Overall survival		Lung cancer-specific survival	
	Hazard ratio^a^ (95% CI)	*P*	Hazard ratio^a^ (95% CI)	*P*
T1N0	0.622 (0.481, 0.804)	0.001	0.600 (0.442, 0.814)	0.001
T2N0	0.625 (0.460, 0.849)	0.003	0.623 (0.445, 0.873)	0.006
T3N0	1.447 (0.832, 2.518)	0.191	1.218 (0.644, 2.302)	0.544
T1-2N1	1.321 (0.954, 1.828)	0.093	1.419 (1.003, 2.006)	0.048

*SCLC* small cell lung cancer, *CI* confidence interval

^a^Hazard ratios were adjusted for age, year of diagnosis, sex, laterality of tumor location, and race

### Comparison between surgery plus postoperative radiotherapy (PORT) and surgery in T1-2N0 cases

**Fig. 4:**
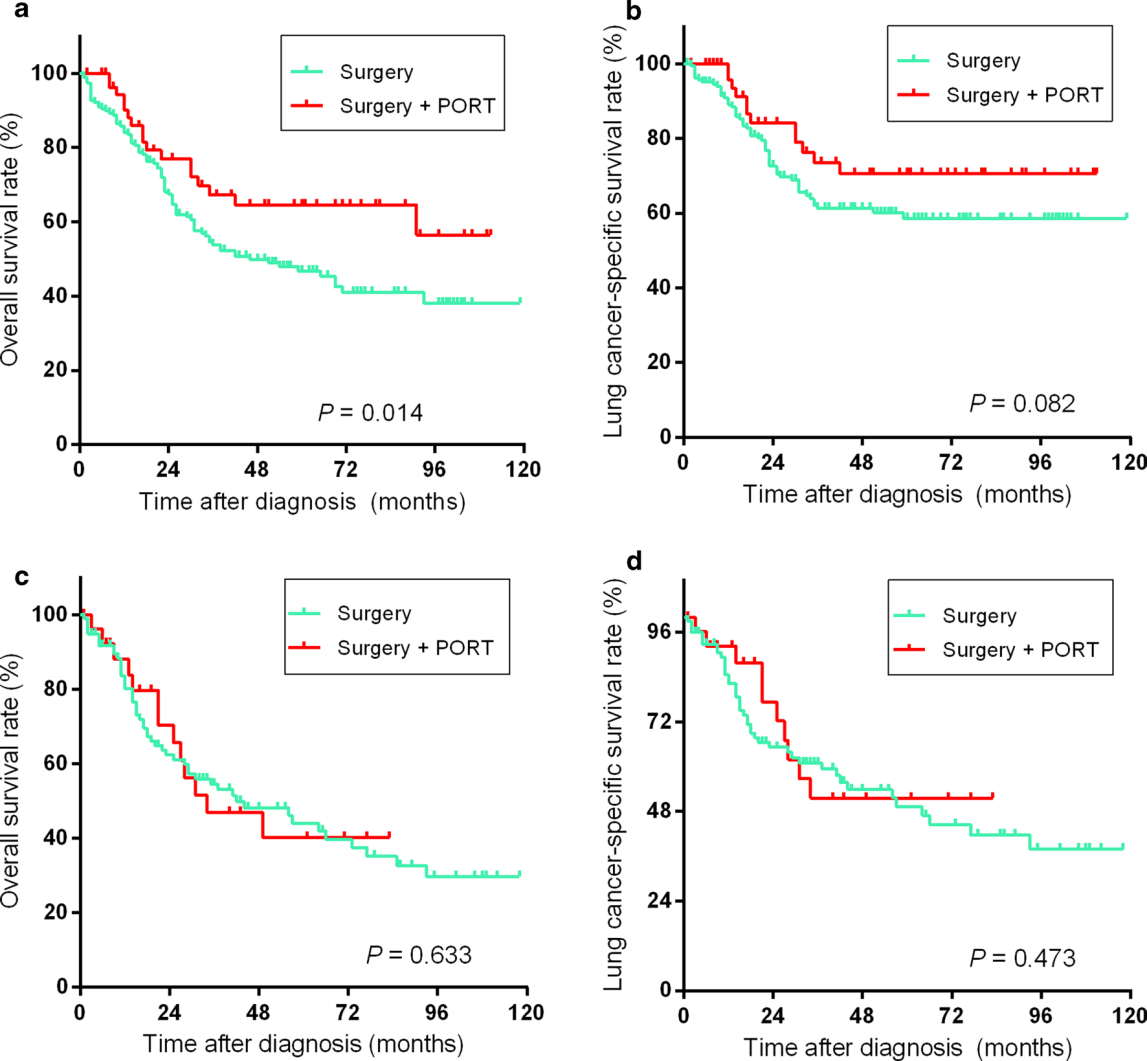
Overall survival (**a**) and lung cancer-specific survival (**b**) for patients with T1N0 SCLC who underwent surgery or surgery + postoperative radiotherapy (PORT) and overall survival (**c**) and lung cancer-specific survival (**d**) for patients with T2N0 SCLC who underwent surgery or surgery + PORT

Among 83 patients with T1-2N0 SCLC who underwent surgery and radiotherapy, 79 (95.2%) patients underwent radiotherapy after surgery. For T1N0 cases, patients who received surgery + PORT had a higher 5-year OS rate than patients who underwent surgery alone (67.8% vs. 46.0%, *P* = 0.014, Fig. 4a), whereas the difference in 5-year LCSS rate between the two groups was not statistically significant (72.1% vs. 58.4%, *P* = 0.082, Fig. 4b). For T2N0 cases, no significant differences in survival rates were found between patients who underwent surgery + PORT and those who underwent surgery alone (5-year OS rate: 49.5% vs. 42.6%, *P* = 0.633, Fig. 4c; 5-year LCSS rate: 54.9% vs. 48.8%, *P* = 0.473, Fig. 4d). The multivariate analysis revealed that after adjustment for age, sex, race, laterality of tumor location, year of diagnosis, and type of resection, the HR for receiving surgery + PORT or surgery alone were not statistically significant in both T1N0 and T2N0 cases in terms of OS (HR: 0.594, 95% CI 0.338–1.044 [T1N0]; HR: 0.956, 95% CI 0.502–1.819 [T2N0]) or LCSS (HR: 0.679, 95% CI 0.349–1.323 [T1N0]; HR: 0.799, 95% CI 0.378–1.689 [T2N0]).

## Discussion

In this large national database-based study, it was found that surgery was associated with longer survival in patients with T1-2N0 SCLC, when compared with radiotherapy. For patients with T3N0 or T1-2N1 SCLC, surgery was associated with shorter survival, compared with radiotherapy, but the difference was not statistically significant. In other words, only patients with T1-2N0 SCLC may benefit from surgery as local treatment.

Chemotherapy is essential in the management of SCLC at present, even for patients with “limited” disease. This recommended therapy scheme was supported by two randomized trials, which found no survival benefits for groups that added surgery to multimodality management for limited stage SCLC [7, 22]. However, these two previous trials suffered critiques due to limitations such as the lack of platinum-based chemotherapy, the presence of bulky nodal disease, and the considerable proportion of patients who received incomplete resection. In addition, peripheral nodules and normal bronchoscopies were excluded in Lad et al.’s study [22], and these nodules might all belong to T1-2 lesions without positive lymph nodes, which was considered to be suitable for surgery in the present study.

In contrast with the conclusions of these two trials, it has been reported that patients with early-stage disease might benefit from surgery [9–18]. The main conclusions of these studies are listed in Table 4.

**Table 4:** Studies that support surgery as part of the multimodality management for selected patients with SCLC

First author and year of publication	Recruitment period	Indications of surgery as local therapy		
		VALG or TNM stage	Tumor size	N category
Rostad, 2004 [12]	1993–1999	I^a^ (T1-2N0, 6th or earlier edition)	Any size	N0
Lim, 2008 [15]	1980–2006	I–II (6th or earlier edition)	Any size	N0
Hanagiri, 2009 [16]	1980–2004	I and part^b^ of II-IIIa	Any size	N0-2
Yu, 2010 [14]	1988–2004	I (T1-2N0, 6th or earlier edition)^c^	Any size	N0
Schreiber, 2010 [9]	1998–2002	Limited stage (T1-4, Nx-0)	Any size	N0
Weksler, 2012 [18]	1988–2007	I-II (6th or earlier edition)	Any size	N0-1
Luchtenborg, 2014 [11]	1998–2009	Early stage based on similar principles for NSCLC	Any size	N0-1
Combs, 2015 [17]	1998–2011	I-III (6th or 7th edition)	Any size	N0-2
NCCN Guideline [19]		T1-2N0 (7th edition)	0–7 cm	N0
The present study	2004–2013	T1-2N0 (8th edition)	0–5 cm	N0

*SCLC* small cell lung cancer, *VALG* Veterans Administration Lung Study Group, *NSCLC* non-small cell lung cancer, *NCCN* National Comprehensive Cancer Network

^a^Patients with stage I lesion located at the peripheral site

^b^Patients with stage II–IIIa SCLC who responded to induction chemotherapy

^c^Patients with stage I SCLC who underwent lobectomy and had reasonable overall survival outcomes

Several single-institution retrospective studies reported that surgery was related to a reasonable survival, with a 5-year OS rate ranging from 45% to 58% for patients with stage I disease [10, 12, 13]. A study based on the SEER database was conducted by Yu et al. [14]. They concluded that patients with stage I SCLC who received lobectomy had a reasonable survival (5-year OS rate: 49.1%). The patients included in these studies were all treated before 2009, when stage I was defined as T1-2N0, which might contain tumors of any size without positive lymph nodes according to the sixth or earlier edition of the TNM classification. Although we also consider that patients with T1-2N0 SCLC were candidates for surgery as local therapy, the present study included less patients (tumor size ≤ 50 mm without positive lymph nodes) than did previous studies.

Other studies concluded that patients with limited stage SCLC, and not only stage I disease, might consider surgery as part of multimodality treatment. Lim et al. [15] identified 59 patients who underwent complete resection and nodal dissection, in which 13 patients had stage II SCLC and 10 patients had stage III SCLC, and the 5-year survival rate for these patients was 52%. Hanagiri et al. [16] reviewed 31 patients treated after 1994 which contained nine patients with stage IIIA SCLC and seven with stage IIIB disease, and a 5-year survival rate of 38.3% was acquired for these patients. The studies above lacked of control groups and did not prove that patients with tumors larger than 50 mm could benefit from surgery [10, 12–16].

Other two studies that included control groups supported that surgery should be considered for patients with, but not limited to, stage I SCLC [17, 18]. A study conducted by Combs et al. [17] identified 2476 patients with resected SCLC from the National Cancer Database. After adjustment for age, stage, and comorbidity scores, it was concluded that the addition of surgery to chemotherapy was associated with a decreased likelihood of death (HR: 0.57, 95% CI 0.47–0.68). The authors concluded that these patients could benefit from surgery, compared with patients in the non-surgery group; however, the authors only compared surgery group with non-surgery group, and they did not compared surgery group with radiotherapy group [17]. Weksler et al. [18] identified 3556 patients with stage I or II SCLC from the SEER database. Pulmonary resection was performed in 895 (25.1%) patients. The median survival was 38.0 months vs. 16.0 months (*P* < 0.001) in patients with stage I disease and 25 months vs. 14 months (*P* < 0.001) in patients with stage II disease in the surgery and non-surgery groups. Similar with the study conducted by Combs et al. [17], the study conducted by Weksler et al. [18] did not distinguish patients who received radiotherapy from the non-surgery group either. Weksler et al. [18] reported that radiotherapy decreased the risk of death by 42% (HR: 0.585, 95% CI 0.537–0.636), whereas surgery decreased the risk by 55% (HR: 0.447, 95% CI 0.389–0.513). Patients benefiting more from surgery than from non-surgery do not mean that surgery may result in longer survival, compared with radiotherapy.

Weksler et al. [18] also compared wedge resection to radiotherapy for patients with stage I or II SCLC, and found that wedge resection prolonged median survival from 16 to 25 months (*P* < 0.001). However, Weksler’s study took patients with stage I or II SCLC as an entirety. When patients with stage I or II SCLC were considered as an entirety, the present study also observed that these patients could benefit from surgery, compared with radiotherapy. However, when the comparison was performed in patients with IIB (T3N0 or T1-2N1) SCLC, the advantage of surgery disappeared.

For patients with T1-2N0 SCLC, who should consider surgery as local therapy, the present study also compared surgery + PORT and surgery alone (Fig. 4). For T1N0 cases, the survival analysis for OS demonstrated that the patients who underwent surgery + PORT had a significantly longer OS than the patients who underwent surgery alone (Fig. 4a); however, neither the survival analysis for LCSS nor multivariable Cox regression revealed that patients with T1N0 could benefit from the addition of PORT to surgery. For T2N0 cases, survival analysis and multivariable Cox regression also demonstrated that patients with T2N0 SCLC could not benefit from the addition of PORT to surgery.

Wong et al. [23] found that the use of PORT deteriorated OS in patients with pN0 disease. Varlotto et al. [24] reported that the addition of irradiation to resection provided no additional benefit for patients with stage I SCLC. Similar with previous studies, the present study also found that T2N0 patients would not benefit from PORT. The multivariate Cox regression analysis results of OS and LCSS and Kaplan–Meier analysis results of LCSS also revealed that T1N0 cases would not benefit from PORT. However, the Kaplan–Meier analysis of OS of T1N0 cases revealed a contradictory outcome. This might be caused by differences in other factors between surgery + PORT and surgery alone in T1N0 patients, since the multivariable analysis results of OS in T1N0 patients revealed that surgery + PORT versus surgery alone was not a significant prognostic factor (HR: 0.594; 95% CI 0.338–1.044). Another potential reason might be the difference in physical status between patients who received surgery + PORT or surgery alone. Patients who underwent surgery + PORT might have a better physical status or pulmonary function than patients who underwent surgery alone. Better physical status might contribute to longer OS for patients who received surgery + PORT. When comparing LCSS, the patients who underwent surgery + PORT no longer had significant longer survival than did those who underwent surgery alone (*P* = 0.082), which might support that the OS benefits of surgery + PORT might be the result of the better physical status of patients who received surgery + PORT.

In addition to treatment, the multivariate analysis also demonstrated that age, year of diagnosis, T category, and N category were independent predictors for mortality. The international association for the study of lung cancer (IASLC) staging project identified that survival was associated with both T category and N category, while no significant difference was observed between cN0 and cN1 [25]. In the present study, we found that N1 did increase the likelihood of overall death and lung cancer-specific death in stage I or II patients (Table 2). Varlotto et al. [24] reported that early year of diagnosis, older age, stage II, and large tumor size were risks of poor survival in patients with stage I or II SCLC who received surgery and/or radiotherapy. However, they did not take the N category into the multivariate model. Tumor size was an independent risk factor for poor survival, which supports that T category was an independent predictor for mortality. In the SEER database analysis, Lally et al. [26] demonstrated that older age, male sex, African American, and larger tumor size were associated with short survival. However, they did not determine whether patients received surgery when constructing the multivariate model. Two other studies from the SEER database also considered older age a hazard factor for survival [9, 18]. Consistent with the multivariate analysis, we observed that patients diagnosed during 2007–2010 and late 2011 had longer OS than patients diagnosed during 2004–2006 (*P* < 0.001, *P* = 0.001). Prolonged survival in patients diagnosed in later years might be due to better cancer detection techniques and staging [27, 28], although we did not find significant differences in T and N categories among patients diagnosed during the periods of 2004–2006, 2007–2009, and late 2010.

In the present study, we did not have access to the chemotherapy data of patients from the SEER database, which may result in bias in the present findings. We could only assume that an overwhelming majority of these patients who could tolerate surgery or radiotherapy received some types of systemic therapy. Bias in staging was another limitation of the present study. Patients who did not receive surgery did not receive pleural invasion examinations and might have been incorrectly staged as IA (since pleura invasion was classified into T2b). Patients in the radiotherapy group might also have occult lymph node metastasis due to the lack of pathologic lymph node staging. Furthermore, TNM stage of patients in the radiotherapy group might be higher than that of patients in surgery group, which would make an adverse impact on survival of patients undergoing radiotherapy. The limitations of the present study also included the inherent bias of the retrospective analysis, and the absence of the comorbidity information and performance status of patients. Patients who received surgery might suffer less comorbidities and have a better performance status than patients who received radiotherapy, which could lead to longer survival for patients in the surgery group. The biases mentioned above, which might benefit patients in the surgery group, indicate that surgery, as an optimal local therapy for T1-2N0 SCLC, required more verifications by prospective studies, but had less impact on the conclusion that patients with T3N0 or T1-2N1 SCLC should consider radiotherapy as local therapy.

## Conclusions

Base on the assumption that the majority of these stage I or II SCLC patients who underwent surgery or radiotherapy also received some types of systemic therapy, the present study recommends that only patients with T1-2 (tumor size ≤ 50 mm) N0 SCLC should consider surgery as local therapy. Patients with T3N0 or T1-2N1 SCLC might consider radiotherapy as local therapy.

## Abbreviations

SCLC: small cell lung cancer
SEER: Surveillance, Epidemiology, and End Results database
RT: radiotherapy
PORT: postoperative radiotherapy
CI: confidence interval
HR: hazard ratio
MRC: Medical Research Council
NCCN: National Comprehensive Cancer Network
MST: median survival time
OS: overall survival
LCSS: lung cancer-specific survival

## References

[1] Chen W, Zheng R, Baade PD (2016). Cancer statistics in china, 2015. CA Cancer J Clin.

[2] Dubey AK, Gupta U, Jain S (2016). Epidemiology of lung cancer and approaches for its prediction: a systematic review and analysis. Chin J Cancer.

[3] Argiris A, Murren JR (2001). Staging and clinical prognostic factors for small-cell lung cancer. Cancer J.

[4] Siegel RL, Miller KD, Jemal A (2017). Cancer statistics, 2017. CA Cancer J Clin.

[5] Simon GR, Wagner H, American College of Chest P (2003). Small cell lung cancer. Chest.

[6] Jett JR, Schild SE, Kesler KA (2013). Treatment of small cell lung cancer: diagnosis and management of lung cancer, 3rd ed: American college of chest physicians evidence-based clinical practice guidelines. Chest.

[7] Fox W, Scadding JG (1973). Medical research council comparative trial of surgery and radiotherapy for primary treatment of small-celled or oat-celled carcinoma of bronchus. Ten-year follow-up. Lancet.

[8] Pignon JP, Arriagada R, Ihde DC (1992). A meta-analysis of thoracic radiotherapy for small-cell lung cancer. N Engl J Med.

[9] Schreiber D, Rineer J, Weedon J (2010). Survival outcomes with the use of surgery in limited-stage small cell lung cancer: should its role be re-evaluated?. Cancer.

[10] Brock MV, Hooker CM, Syphard JE (2005). Surgical resection of limited disease small cell lung cancer in the new era of platinum chemotherapy: its time has come. J Thorac Cardiovasc Surg.

[11] Luchtenborg M, Riaz SP, Lim E (2014). Survival of patients with small cell lung cancer undergoing lung resection in england, 1998–2009. Thorax.

[12] Rostad H, Naalsund A, Jacobsen R (2004). Small cell lung cancer in norway. Should more patients have been offered surgical therapy?. Eur J Cardiothorac Surg Off J Eur Assoc Cardiothorac Surg.

[13] Nakamura H, Kato Y, Kato H (2004). Outcome of surgery for small cell lung cancer—response to induction chemotherapy predicts survival. Thorac Cardiovasc Surg.

[14] Yu JB, Decker RH, Detterbeck FC (2010). Surveillance epidemiology and end results evaluation of the role of surgery for stage i small cell lung cancer. J Thorac Oncol Off Publ Int Assoc Study Lung Cancer.

[15] Lim E, Belcher E, Yap YK (2008). The role of surgery in the treatment of limited disease small cell lung cancer: time to reevaluate. J Thorac Oncol Off Publ Int Assoc Study Lung Cancer.

[16] Hanagiri T, Sugio K, Baba T (2009). Results of surgical treatment for patients with small cell lung cancer. J Thorac Oncol Off Publ Int Assoc Study Lung Cancer.

[17] Combs SE, Hancock JG, Boffa DJ (2015). Bolstering the case for lobectomy in stages i, ii, and iiia small-cell lung cancer using the national cancer data base. J Thorac Oncol Off Publ Int Assoc Study Lung Cancer.

[18] Weksler B, Nason KS, Shende M (2012). Surgical resection should be considered for stage i and ii small cell carcinoma of the lung. Ann Thorac Surg.

[19] NCCN National Comprehensive Cancer Network I. National comprehensive cancer network guidelines for small cell lung cancer version 3.2017. 2017. http://www.nccn.org/professionals/physician_gls/pdf/sclc.pdf. Accessed 1 Mar 2017.

[20] Nicholson AG, Chansky K, Crowley J (2016). The international association for the study of lung cancer lung cancer staging project: proposals for the revision of the clinical and pathologic staging of small cell lung cancer in the forthcoming eighth edition of the tnm classification for lung cancer. J Thorac Oncol Off Publ Int Assoc Study Lung Cancer.

[21] Rami-Porta R, Bolejack V, Crowley J (2015). The iaslc lung cancer staging project: proposals for the revisions of the t descriptors in the forthcoming eighth edition of the tnm classification for lung cancer. J Thorac Oncol Off Publ Int Assoc Study Lung Cancer.

[22] Lad T, Piantadosi S, Thomas P (1994). A prospective randomized trial to determine the benefit of surgical resection of residual disease following response of small cell lung cancer to combination chemotherapy. Chest.

[23] Wong AT, Rineer J, Schwartz D (2016). Assessing the impact of postoperative radiation therapy for completely resected limited-stage small cell lung cancer using the national cancer database. J Thorac Oncol Off Publ Int Assoc Study Lung Cancer.

[24] Varlotto JM, Recht A, Flickinger JC (2011). Lobectomy leads to optimal survival in early-stage small cell lung cancer: a retrospective analysis. J Thorac Cardiovasc Surg.

[25] Shepherd FA, Crowley J, Van Houtte P (2007). The international association for the study of lung cancer lung cancer staging project: proposals regarding the clinical staging of small cell lung cancer in the forthcoming (seventh) edition of the tumor, node, metastasis classification for lung cancer. J Thorac Oncol Off Publ Int Assoc Study Lung Cancer.

[26] Lally BE, Geiger AM, Urbanic JJ (2009). Trends in the outcomes for patients with limited stage small cell lung cancer: an analysis of the surveillance, epidemiology, and end results database. Lung cancer.

[27] Pieterman RM, van Putten JW, Meuzelaar JJ (2000). Preoperative staging of non-small-cell lung cancer with positron-emission tomography. N Engl J Med.

[28] Henschke CI, Yankelevitz DF, International Early Lung Cancer Action Program I (2006). Survival of patients with stage i lung cancer detected on ct screening. N Engl J Med.

